# Dependence of Shape-Based Descriptors and Mass Segmentation Areas on Initial Contour Placement Using the Chan-Vese Method on Digital Mammograms

**DOI:** 10.1155/2015/349874

**Published:** 2015-08-24

**Authors:** S. N. Acho, W. I. D. Rae

**Affiliations:** Department of Medical Physics, University of the Free State, P.O. Box 339, Bloemfontein 9300, South Africa

## Abstract

Variation in signal intensity within mass lesions and missing boundary information are intensity inhomogeneities inherent in digital mammograms. These inhomogeneities render the performance of a deformable contour susceptible to the location of its initial position and may lead to poor segmentation results for these images. We investigate the dependence of shape-based descriptors and mass segmentation areas on initial contour placement with the Chan-Vese segmentation method and compare these results to the active contours with selective local or global segmentation model. For each mass lesion, final contours were obtained by propagation of a proposed initial level set contour and by propagation of a manually drawn contour enclosing the region of interest. Differences in shape-based descriptors were quantified using absolute percentage differences, Euclidean distances, and Bland-Altman analysis. Segmented areas were evaluated with the area overlap measure. Differences were dependent upon the characteristics of the mass margins. Boundary moments presented large percentage differences. Pearson correlation analysis showed statistically significant correlations between shape-based descriptors from both initial locations. In conclusion, boundary moments of digital mass lesions are sensitive to the placement of initial level set contours while shape-based descriptors such as Fourier descriptors, shape convexity, and shape rectangularity exhibit a certain degree of robustness to changes in the location of the initial level set contours for both segmentation algorithms.

## 1. Introduction

Breast masses are one of the most common indications of breast cancer. They are frequently identified on mammograms, due to their saliency relative to the surrounding regions and also to comparable regions on the mammograms with the same projection of the opposite breast [1]. Computer Aided Detection algorithms for breast mass classification exploit suitable shape-based descriptors derived from the mass boundary which are powerful enough to differentiate between benign and malignant masses. Segmentation algorithms are necessary for mass contouring in direct digital mammography. However, in this imaging modality, mass margins are embedded in complex backgrounds of overlying and underlying tissues which creates missing boundary information and local minima where a deforming contour can be entrapped and as a consequence produces an undesirable segmentation outcome. Moreover, the wide dynamic range of flat panel detector systems of direct digital mammography units records small differences between the attenuation coefficients of structures or regions present in a mass lesion and they are clearly distinguishable over a wide range of densities, whereas in film screen mammography the exposure latitude of the film limits the dynamic range of information captured on the film. Hence, masses which may have appeared as dense structures without significant topographical relief features on film screen mammograms can emerge following digital imaging, as regions with varying densities on soft copy display. Enhancement of these variations, following postprocessing by the processing algorithms of the manufacturer, may also be present. Usually, small differences in densities may sometimes appear as low signal areas which can act as local minima for contour entrapment each time an evolving curve determines its path within the mass lesion. Consequently, local minima and missing boundary information render deformable contours susceptible to their initial locations.

A geometric active contour is a deformable contour based approach for image segmentation. In breast mass segmentation, an initial contour is deformed and driven by a partial differential equation (PDE) towards the boundary of the candidate mass. It is categorized into two groups: edge based models [2–4] and region based models [5–13]. Both models make use of a stopping term which reduces the speed of the evolving contour as it approaches the boundary of the object and finally reaches a steady state at the boundary. In edge based models, the stopping term utilizes an edge indicator function modelled on the image gradient; consequently, objects with weak and noisy boundaries may present some difficulties to this segmentation model [14, 15].

The Chan-Vese region based algorithm models energy functionals as a competition of regional statistical information [16]. They defined the stopping term as a competition of the first moments of the local intensity distribution of the foreground and the background within a narrowband, which takes into consideration only pixels which will influence the propagation of the interface (zero level set function) between these two regions. The energy functionals drive the initial contour from its initial location toward a desirable local minimum, which in principle should correspond to the delineated boundary of an expert radiologist. However, these are determined by localized statistics; hence, the evolution of the curve becomes sensitive to the location of the initial level set contour and segmentation results will depend on the placement of this contour, especially when tuning parameters for an arbitrary collection of masses are fixed. This becomes evident during segmentation of direct digital masses with obscured or ill-defined margins and low signal areas within.

The active contours with selective local or global segmentation model [9] are a region based energy functional formulated as a signed pressure force function which propagates the initial contour by modulating the signs of the pressure forces inside and outside the region of interest. These pressure forces are derived from the means of the local intensity distributions of the foreground and the background. The algorithm penalizes the level set function to be binary and regularizes it with a Gaussian smoothing kernel. It can effectively handle images with weak edges and interior intensity inhomogeneity.

In most segmentation problems, the initial contour is either drawn by the operator or estimated from other segmentation algorithms [17, 22–25] and this may place the initial level set contour on different locations within the mass. Any variation in segmentation outcomes will cause changes in shape-based descriptors and the area occupied by the segmented mass. Variations in segmentation outcomes which are due to the placement of the initial level set contours in complicated images have been mentioned [11]. Mass lesions on mammograms are complicated image domains for curve evolution and variations in mass lesion segmented areas and their influences on shape-based feature vectors due to changes in the placement of the initial level set contours are not found in the literature.

Understanding these inconsistencies can improve the choice of tuneable parameters and initial contour locations for curve evolution either for a data set of mass lesions with labelled margin characteristics or unlabelled margin characteristics. Shape-based descriptors [26–28] are feature vectors in training sets for binary classification of mass lesions in mammography and changes in these descriptors can play a role in determining the interclass separability measures, the choice of margin hyperplanes, and hence the classification efficiencies of these algorithms.

In this study, we investigate changes in one-dimensional shape-based descriptors and the segmented areas of masses in direct digital mammograms due to changes in the location of the initial level set contours with the implementation of the Chan-Vese segmentation method and the active contours with selective local or global segmentation model. Two groups of masses are considered in this study, one with obscured or ill-defined margins and low signal areas within and the other with well-defined and distinct margins. We consider a contour which encloses the mass lesion and is propagated towards the margin of the lesion. We propose a semiautomatic method which derives the initial contour as a curve connecting points with maximum gradient in the radial direction, representing an optimum curve characterizing the intrinsic shape of the mass lesion, and then assess the differences in the segmentation results.

## 2. Background to Mathematical Methods

In mammography, smoothed images present topological surfaces that can be thresholded into multiple layers to obtain topographical relief maps of dominant structures found on the images. Mammograms are filtered with edge-preserving denoising methods such as weighted total variation (TV) scale-space smoothing technique [29, 30] to remove noise and fine details while preserving dominant edge characteristics through different degrees of smoothing.

### 2.1. Weighted Total Variation Scale-Space Smoothing Technique

Suppose *I* : Ω → *ℝ* denotes an image and Ω ⊂ *ℝ* ^2^ the image domain. The variational approach for image denoising for this model involves the minimization of the following energy functional:

(1) $$\begin{array}{c} E_{TV}\left(\;I,\lambda \;\right)=\int_{\Omega }\left(\;\left|\;\nabla I\;\right|+\lambda {\left(\;I-I_{0}\;\right)}^{2}\;\right)dx\;dy, \end{array}$$

where *I*_0_ is the noisy input image and *I* its regularized approximation. *λ* is the Lagrange multiplier indicating the scale of detail desired in the smoothed image. Bresson et al. proposed a modified model [30] in which the *L* ^2^-norm square of Rudin et al.'s model is replaced with an *L* ^1^-norm to preserve image contrast [31] and in addition the TV norm of *I* is multiplied with a function, *g*, which is an edge indicator function. This represents the weighted TV model with an *L* ^1^-norm as a data fidelity measure. The energy functional for minimization is given as

(2) $$\begin{array}{c} E_{gTV}\left(\;I,\lambda \;\right)=\int_{\Omega }\left(\;g\left|\;\nabla I\;\right|+\lambda \left|\;I-I_{0}\;\right|\;\right)dx\;dy \end{array}$$

with

(3) $$\begin{array}{c} g=\frac{1}{{1+\Upsilon \left|{\nabla G}_{\theta }*I_{0}\right|}^{2}}, \end{array}$$

where *Υ* is a constant >0 and *G*_*θ*_ is a Gaussian kernel with standard deviation, *θ*. The minimization of *E*_*g*TV_(*I*, *λ*) results in the following weighted TV flow equation:

(4) $$\begin{array}{c} I_{t}=\mathrm{div}\left(\;g\frac{\nabla I}{\left|\nabla I\right|}\;\right)+\lambda \left(\;\frac{I-I_{0}}{\left|I-I_{0}\right|}\;\right). \end{array}$$

For small values of *λ*, the degree of image smoothing increases and edge is preserved; therefore, the global boundary information which is essential for segmentation algorithms can be modelled as the initial contour for the gradient descent flow equation of the level set. This contour will depend on the boundary properties of a given mass lesion.

### 2.2. Chan-Vese's Piecewise Constant Model for Binary Segmentation

Suppose *C* is an evolving curve that partitions the image domain into the foreground, Ω_1_, and the background, Ω_2_. The Chan-Vese model [16] seeks an optimal contour, representing the boundary of an object by minimizing the following energy functional:

(5) $$\begin{array}{c} F\left(\;C,c_{1},c_{2}\;\right)=\mu \text{length}\left(\;C\;\right)+\upsilon \text{Area}\left(\;\text{inside}\left(\;C\;\right)\;\right)+F_{data}, \end{array}$$

where *F*_data_ represents the regional term guiding the contour in the image domain and is given by

(6) $$\begin{array}{c} F_{data}=\lambda_{1}F_{1}\left(\;C\;\right)+\lambda_{2}F_{2}\left(\;C\;\right) \end{array}$$

in which

(7) F1C=∫insideCIx,y−c12dx dy,F2C=∫outsideCIx,y−c22dx dy.

*μ* ≥ 0, *ν* ≥ 0, and *λ*_1_ and *λ*_2_ are positive constants while the average image intensities of regions inside and outside the contour are *c*_1_ and *c*_2_, respectively. In level set formulation, the interface of the foreground and background is embedded as the zero level set of a Lipschitz function, *ϕ*(*x*, *y*): Ω → *ℝ* with *ϕ*(*x*, *y*) > 0 for pixel positions in *Ω*_1_ and *ϕ*(*x*, *y*) < 0 for pixel positions in *Ω*_2_ whilst *ϕ*(*x*, *y*) = 0 on the curve *C*. Using the Heaviside step function, *H*_*ε*_(*ϕ*), *F*(*C*, *c*_1_, *c*_2_) can be expressed as

(8) FC,c1,c2=μ∫Ω∇Hεϕdx dy+λ1∫ΩIx,y−c12Hεϕdx dy+λ2∫ΩIx,y−c221−Hεϕdx dy.

Minimizing *F*(*C*, *c*_1_, *c*_2_) with respect to *ϕ* yields the following gradient descent flow:

(9) ∂ϕ∂t=δεϕμ∇∇ϕ∇ϕ−υ−λ1Ix,y−c12+λ2Ix,y−c22,

where *δ*_*ε*_(*ϕ*) is the Dirac function.

### 2.3. Active Contours with Selective Local or Global Segmentation Model

The signed pressure force function [9] is derived from the means of regions inside and outside the contour and it is defined as

(10) spfIx,y=Ix,y−c1+c2/2max⁡Ix,y−c1+c2/2,x,y∈Ωp,

where *c*_1_ and *c*_2_ are defined in (8). The active contour with selective local or global segmentation model utilizes the geodesic active contour to formulate the level set equation as

(11) ∂ϕ∂t=spfIx,y·div⁡∇ϕ∇ϕ+α∇ϕ+∇spfIx,y·∇ϕ,x,y∈Ωp.

Using the Gaussian filtering process to regularize the level set function, the above equation can be written as follows:

(12) $$\begin{array}{c} \frac{\partial \phi }{\partial t}=\text{spf}\left(\;I\left(\;x,y\;\right)\;\right)\cdot \alpha \left|\;\nabla \phi \;\right|,\;x,y\in \Omega_{p}, \end{array}$$

where *α* is a tuneable parameter.

## 3. Method

### 3.1. Data Set Description

Direct digital mammograms were acquired from a Hologic Selenia Dimensions system with an image receptor consisting of a 70 *μ*m pixel pitch selenium direct-capture detector. Ninety mammograms with mass lesions were selected for this study. Forty mammograms had masses with low signal areas within the mass and margins described as obscured, or ill-defined, while the others had masses with well-defined or distinct margins. On each mammogram, the region of interest containing the mass lesion was cropped and then resized to a 208 × 208 matrix to create a submammogram. Each submammogram was denoised and thresholded to localize the initial level set contour.

### 3.2. Search Space for Localizing the Initial Level Set Contour

The weighted total variation scale-space smoothed breast mass region is represented as a topological surface in which the grey level value of each pixel is the height of the surface. Let *IS* : *Ω* → *ℝ* denote a smoothed image and *Ω* ⊂ *ℝ* ^2^ the image domain. The image domain *Ω* is thresholded into multiple regions with an ordered set of equally spaced grey level threshold values within the intensity range of the image domain [32–34]. Suppose *I*_max_ = the maximum grey level intensity in the image domain; *I*_min_ = minimum grey level intensity; *W* = {*w*_1_, *w*_2_, *w*_3_,…, *w*_*N*_}, a finite sequence of equally spaced partition weights in ascending order; *N* = number of threshold values; and *T* = {*t*_1_, *t*_2_, *t*_3_,…, *t*_*N*_}, an ordered set of equally spaced grey level threshold values; then,

(13) $$\begin{array}{c} T=I_{max}*W \end{array}$$

with *t*_*N*_ ≤ *I*_max_ and *t*_1_ ≥ *I*_min_.

The subregions in the image domain with grey level intensities less than or equal to the threshold value, *t*_*i*_, are given as

(14) $$\begin{array}{c} R\left(\;t_{i}\;\right)=\left\{\;\left(\;\left.\;x,y\;\right)\;|\;IS\left(\;x,y\;\right.\;\right)\leq t_{i}\;\right\},\;\forall \left(x,y\right)\in \Omega , \end{array}$$

and the iso-level contours *C*(*t*_*i*_)'s of these regions are boundaries of *R*(*t*_*i*_). The iso-level contour map of the image domain represents the set of all *C*(*t*_*i*_) for *i* = 1 : *N*. A graph-based representation of the iso-level contour map evaluates the enclosure relationship between an iso-level contour and its nearest neighbour, to identify the path to the base contour that delineates the mass. Details of this method can be found in the literature [32, 33]. In our implementation, the boundary region of the breast mass is the region around the base contour with a dense nested pattern of iso-level contours, indicating the search space for the actual boundary of the mass and the placement of the initial level set contour. The dense nested pattern of iso-level contours is extracted and superimposed on the gradient map of the smoothed image.

### 3.3. Placement of the Initial Level Set Contour

**Figure 1:**
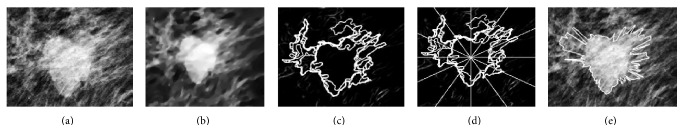
Search space for localizing the initial contour. (a) The original mass lesion, (b) the weighted TV flow denoised image with *λ* = 0.05, (c) dense nested patterns of iso-level contours representing the search space for localizing the initial contour on the gradient map, (d) radial distances from a reference point to the iso-level contours, and (e) initial level set contour, representing points with maximum gradient in the radial direction within a predefined radius.

A set of uniformly spaced radial lines, *L* = {*l*_1_, *l*_2_,…, *l*_*m*_}, are generated from a point close to the centre of mass of the innermost iso-level contour, defining the search space on the gradient map of the mass as shown in Figure 1(d). Let this point be the reference point. The gradient strength is noted at every point of intersection of the nested iso-level contours and radial lines. Along each radial line, *l*_*i*_, for *i* = 1,2,…, *m*, the coordinates of the point of intersection with the greatest gradient strength are noted and the radial distance from this point to the reference point is calculated and noted as *r*_*i*_.

Let *r*_ave_ = (1/*m*)∑_*i*=1_^*m*^ *r*_*i*_ and $r_{std}=\sqrt{\sum_{i=1}^{m}{\left(r_{i}-r_{ave}\right)}^{2}/\left(m-1\right)}$; then radial description of the initial level set contour is given by

(15) ri=ri,ri<rave+nrstdrave,ri≥rave+nrstd,i=1,2,…,m,  n=1  or  2.

**Figure 2:**
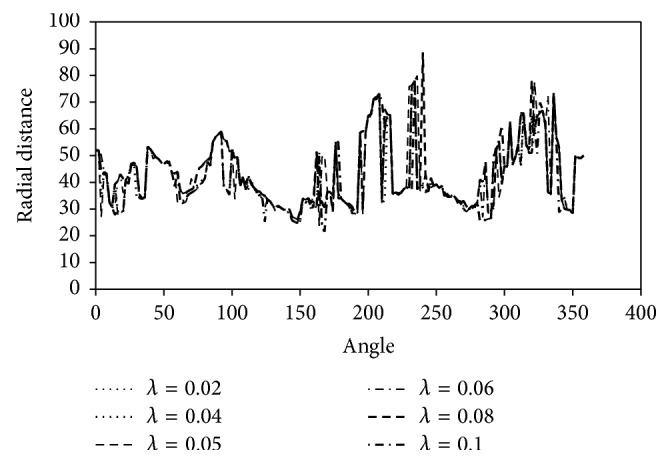
Variation of the radial distance function of the initial level set contours sampled at an angle of 1° with different *λ*'s for the mass lesion in Figure 1.

The spatial coordinates of the points of intersection of *r*_*i*_'s and the iso-level contours are the coordinates of the initial level set contour.  Figure 1 illustrates the summary of the methodology in acquiring the initial level set contour and Figure 2 shows the variation of the radial distance function, *r*_*i*_, for *i* = 1 : *m*, with the scale of observation, *λ*, in weighted total variation scale-space smoothing technique. The radial distance function of the initial level set contour corresponds to the radial distance from each point on the initial contour to the reference point with a sampling angle of 1°.

### 3.4. Evaluation Metrics of Segmentation Results

Manually drawn initial contours and those obtained from our proposed method were propagated with the Chan-Vese algorithm and the active contours with selective local or global segmentation model. Feature vectors representing boundary-based shape signatures and the areas occupied by the segmented mass lesions were assessed to provide relative measures of the differences between the segmented mass lesions.

#### 3.4.1. Area Metric of Relative Size of Segmented Mass Lesion

Let im*Y* represent the binary image obtained by evolving the initial level set contour from our proposed method and im*X* from the manually drawn initial level set contour; then, the area overlap measure, which is the Jaccard similarity coefficient between the binary images, im*X* and im*Y*, is given as

(16) $$\begin{array}{c} \text{JSC}\left(\;\text{im}X,\text{im}Y\;\right)=\frac{\text{im}Y\cap \text{im}X}{\text{im}Y\cup \text{im}X}. \end{array}$$

JSC(im*X*, im*Y*) lies between 0 and 1. A perfect match between im*X* and im*Y* is achieved as JSC(im*X*, im*Y*) → 1, consequently, the same segmentation outcome for both initial level set contours.

#### 3.4.2. Evaluation Metrics of Shape-Based Descriptors

*Boundary Moments.* A boundary-based shape signature of the segmented mass lesion from each initial contour model is represented as the centroid distance function, which is a one-dimensional function representing the Euclidean distance *r*(*n*) between an ordered set of boundary coordinates ((*x*(*n*), *y*(*n*)),  for  *n* = 0,2, 3,…, *N* − 1) and the centroid (*xc*, *yc*) signifying the centre of mass of the binary image generated from the contour:

(17) $$\begin{array}{c} r\left(\;n\;\right)=\sqrt{\left({\left(x\left(n\right)-xc\right)}^{2}+{\left(y\left(n\right)-yc\right)}^{2}\right)}, \end{array}$$

where *N* is the total number of points on the contour.

The centroid distance function captures the local and global characteristics of the final shape of the segmented mass lesion. Its statistical characteristics are assessed as shape features derived from the contour sequence moments *m*_*p*_ and *μ*_*p*_ [35] where the *p*th contour sequence moment is estimated as

(18) $$\begin{array}{c} m_{p}=\frac{1}{N}\overset{N-1}{\underset{n=0}{\sum }}{\left[\;r\left(\;n\;\right)\;\right]}^{p} \end{array}$$

and the *p*th central moment is estimated as

(19) $$\begin{array}{c} \mu_{p}=\frac{1}{N}\overset{N-1}{\underset{n=0}{\sum }}{\left[\;r\left(\;n\;\right)-m_{1}\;\right]}^{p}. \end{array}$$

These shape features are normalized low-order boundary moments [36, 37] described as

(20) F1=μ21/2m1,F2=μ41/4m1,F3=F1−F2,

where *F*_1_ is the normalized amplitude variation and *F*_2_ and *F*_3_ are indicators of shape roughness.

Spicules are fine extensions radiating from the margin of a mass lesion. The presence of these boundary features generates variations in the radial distances, which are indicative of contour roughness along the boundary of a mass lesion. The evaluation metric %Δ*F*_*i*_(im*X*, im*Y*) is the percentage change in the degree of spiculation between im*X* and im*Y* and is expressed as the percentage difference in the boundary moments, *F*_*i*_'s:

(21) %ΔFiimX,imY=FiimY−FiimXaverageFiimY,FiimX×100.

*Fourier Descriptors.* The centroid distance function can be analysed in the frequency domain to obtain spectral descriptors of its characteristics. Its spectral representation is expressed as the coefficients of its discrete Fourier transform, yielding

(22) ai=1N∑n=0N−1rnexp⁡−j2πinN,i=0,1,2,…,N−1.

Feature vectors which are invariant to translation, scale, and rotation are extracted from these coefficients and are known as the Fourier descriptors (FD_*i*_) for shape representation:

(23) $$\begin{array}{c} {\text{FD}}_{i}=\left[\;\frac{\left|a_{i}\right|}{\left|a_{0}\right|}\;\right],\;i=1,2,\ldots ,\frac{N}{2}. \end{array}$$

Zhang and Lu [38] have shown that FD_*i*_ derived from the centroid distance function outperforms FD_*i*_'s derived from using complex coordinates, cumulative angles, and curvature function as boundary signatures in shape-based image retrieval system, and furthermore, in Zhang and Lu [39], they mentioned that 60 FD_*i*_'s are sufficient for shape indexing.

We define the evaluation metric of the initial level set contours yielding im*X* and im*Y* based on the boundary signatures of the final contours delineating im*X* and im*Y* in the frequency domain as the Euclidean distance (DF(im*Y*, im*X*)) between the Fourier descriptors of the images:

(24) $$\begin{array}{c} DF\left(\;\text{im}Y,\text{im}X\;\right)=\sqrt{\overset{60}{\underset{i=1}{\sum }}{\left|{\text{FD}}_{i}\left(\text{im}Y\right)-{\text{FD}}_{i}\left(\text{im}X\right)\right|}^{2}}, \end{array}$$

where FD_*i*_(im*X*) and FD_*i*_(im*Y*) are the *i*th Fourier descriptors of the final contours delineating im*X* and im*Y*.

*Shape Convexity.* Shape convexity measures the degree of spiculation in masses. The shape convexity of a binary image is defined as the ratio of the area of the binary image to the area of its convex hull [26]. Let *C*im*X* and *C*im*Y* be the convexity of binary images im*X* and im*Y*, respectively; the evaluation metric of the difference between the shape convexities of images im*X* and im*Y* is defined as

(25) %ΔSCimX,imY=CimY−CimXaverageCimY,CimX×100.

*Shape Rectangularity.* Shape rectangularity [40] is defined as the ratio of the area of the binary image to the area of its minimal bounding rectangle. Let *R*im*X* and *R*im*Y* be the shape rectangularity of binary images im*X* and im*Y*, respectively; the evaluation metric of the difference between the shape rectangularities of images im*X* and im*Y* is defined as

(26) $$\begin{array}{c} \%\Delta \text{SR}\left(\;\text{im}X,\text{im}Y\;\right)=\left|\;\frac{R\text{im}Y-R\text{im}X}{\text{average}\left(R\text{im}Y,R\text{im}X\right)}\;\right|\times 100. \end{array}$$

Differences in shape-based descriptors of the final contours were further evaluated with Bland-Altman analysis to explore the agreement and trends between placements of the initial level set contours in digital mass lesions segmentation while Pearson correlation analysis assessed the correlation between these descriptors.

## 4. Experimental Results and Discussion

In our implementation of the Chan-Vese method, we set *μ* = 0.2, *λ*_1_ = 2.5, and *λ*_2_ = 1. We chose *λ*_1_ > *λ*_2_ to give a greater weight to the variance of pixels in the foreground so as to achieve measurable segmentation differences between the proposed locations for the initial level set contours. Furthermore, we assigned *λ*_1_ = *λ*_2_ = 1 to investigate changes in the final segmentation results due to differences in tuneable parameters. In practice, for a given database of masses, the values assigned to *λ*_1_ and *λ*_2_ depend on the similarity indices between segmentation results of a proposed algorithm and the gold standard of a training set of masses, which in some cases is a subset of the database. For the active contour with selective local or global segmentation model, we set *α* = 5 for this database so that masses with ill-defined boundaries should be accurately segmented. The segmentation performances of this algorithm were poor with values of *α* > 5 for this group of masses. The average time for curve evolution for these images was 15 ± 10 s for the segmentation methods.

Boundary information represents sharp changes in image properties. Figure 2 shows that as the degree of smoothing increases the radial distance functions of the initial level set contours form a dense nested pattern of curves. The differences between these curves are very small because edge is preserved through different values of *λ*'s in weighted TV scale-space smoothing technique; consequently, segmentation results with the initial level set contours generated from these curves are expected to be similar.

**Figure 3:**
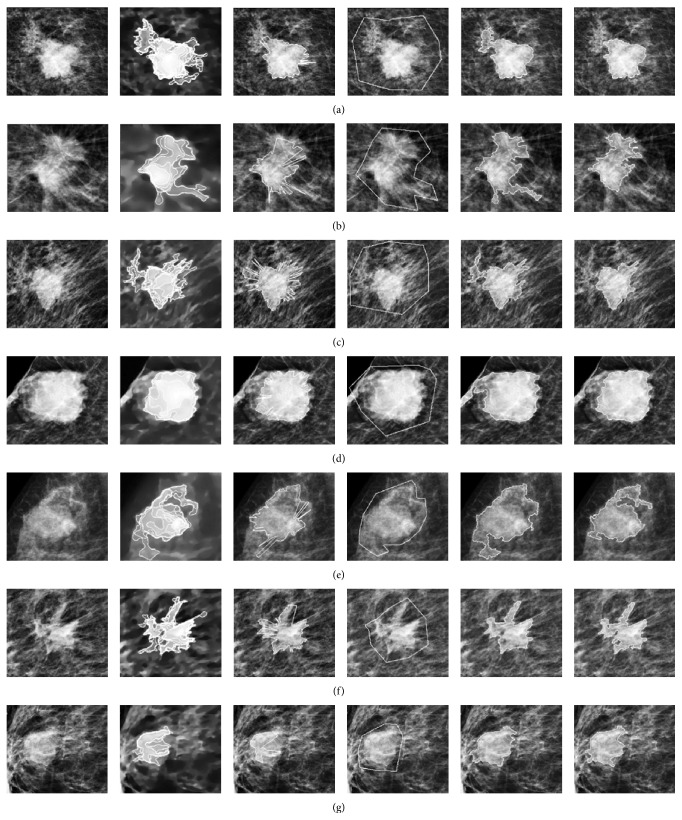
Comparisons of segmentation results with different locations for the initial level set contours for masses with low signal areas having obscured, or ill-defined, margins with the Chan-Vese model. The first column presents the original mass lesions; the second column shows the corresponding weighted TV flow images and the search space for locating the initial contour. The third column shows the initial contours as curves connecting points with maximum gradients in the radial direction. The fourth column shows the manually drawn initial level set contours. The fifth column presents the segmentation outcomes with manually drawn initial level set and the last column presents the final segmentation results of the proposed method evolved with the same tuning parameters.

Segmentation results for some masses with low signal areas and having obscured, or ill-defined, margins are shown in Figure 3. The proposed method defines the initial level set contour as the curve connecting points with maximum gradients in the radial direction as shown in column 3. Each curve characterizes the intrinsic shape of its mass lesion and its evolution is guided by the statistics of pixels surrounding the region. For this group of masses, the mean area overlap measure between segmented areas generated from the final contours of our proposed method and that of the manually drawn initial level set contours were 0.81 ± 0.01 for the Chan-Vese model and 0.86 ± 0.09 with the selective local or global segmentation model. This is almost comparable to the mean area overlap measures between expert radiologists [17] and expert radiologists against segmentation methods [17–21] as shown in Table 1. Therefore, changes in shape-based descriptors as expressed in our setup will be suggestive of changes in shape-based descriptors encountered by the abovementioned publications.

Table 2 shows the variation in the area overlap measures with percentage differences in boundary moments *F*_1_, *F*_2_, and *F*_3_ when masses in Figure 3 were evolved with tuneable parameters *λ*_1_ = 2.5, *λ*_2_ = 1. The area overlap measure of mass D is greater than 0.8; however, the percentage difference in boundary moments was above 50%, with %Δ*F*_1_ being 87.0%. The mean values of %Δ*F*_1_, %Δ*F*_2_, and %Δ*F*_3_ for this group were 23.9% (range 1.0–87.0%), 24.5% (range 1.7–86.8%), and 32% (range 1.4–86.0%), respectively, as shown in Table 6. The mean values are large with wide range. For *λ*_1_ = 1, *λ*_2_ = 1, the mean values of the percentage change of each boundary moment were less than 20.2%. These large ranges and mean values show that boundary moments are sensitive to the location of the initial level set contour for masses with obscured or ill-defined margins and the degree of sensitivity depends on the choice of tuneable parameters. As shown in Table 7, the mean values of boundary moments %Δ*F*_1_, %Δ*F*_2_, and %Δ*F*_3_ were obtained as 15.1% (range 0–74%), 15.4% (range 0–67.5%), and 23.5% (range 0–52%), respectively, by using the selective local or global segmentation model. These values are comparable to values obtained by implementing the Chan-Vese model for *λ*_1_ = 1, *λ*_2_ = 1.

In Table 3, the variation in Euclidean distances of the Fourier descriptors and the percentage differences in shape convexity and rectangularity for the masses in Figure 3 are illustrated. In Table 6, for *λ*_1_ = 2.5, *λ*_2_ = 1, the mean Euclidean distance between the Fourier descriptors of the segmented areas was 0.09 ± 0.05 while the mean values of percentage changes in shape convexity and rectangularity were 8.3% (range 0.0–28.1%) and 11.7% (range 0.1–42.0%), respectively, with more than 50% reduction in the mean values with tuneable parameters *λ*_1_ = 1, *λ*_2_ = 1. The values for the mean percentage difference in shape convexity and rectangularity and their range were less than those from boundary moments for both Chan-Vese algorithms. The selective local or global segmentation model presented similar results for the percentage differences in shape convexity and shape rectangularity as shown in Table 7.

**Figure 4:**
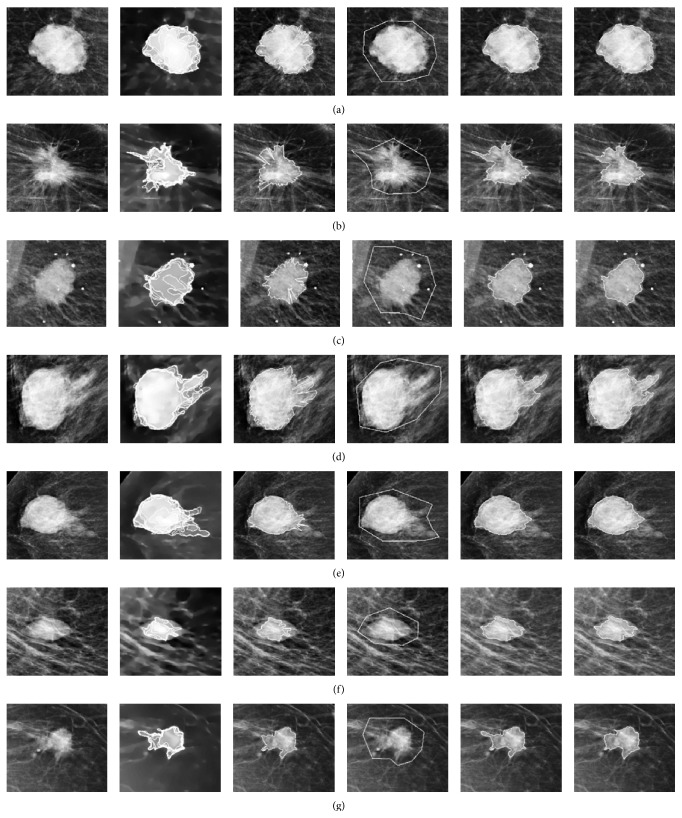
Comparisons of segmentation results with different locations for the initial level set contours for masses with distinct, or well-defined, margins by implementing the Chan-Vese model. The first column presents the original mass lesions; the second column shows the corresponding weighted TV flow images and the search space for locating the initial contour. The third column shows the initial contours as curves connecting points with maximum gradients in the radial direction. The fourth column shows the manually drawn initial level set contours. The fifth column presents the segmentation outcomes with manually drawn initial level set contours and the last column presents the final segmentation results of the proposed method evolved with the same tuning parameters.

Figure 4 illustrates the segmentation results with different locations for the initial level set contours for some masses with distinct, or well-defined, margins. The initial level set contour from the proposed method is shown in column 3. Fewer points defining the maximum gradients in the radial direction are found within the mass lesion, as compared with the previous group. Most points defining the maximum gradients in the radial direction are found on the mass boundary; consequently, the statistics of the pixels surrounding the initial level set contour will be similar to those of the manually drawn contour when it arrives at the edge of the mass lesion.

Table 4 shows the variation in the area overlap measures and the percentage differences in boundary moments *F*_1_, *F*_2_, and *F*_3_ while Table 5 illustrates the variation in Euclidean distances between the Fourier descriptors (DF), percentage differences in shape convexity (%ΔSC), and shape rectangularity (%ΔSR) when the masses in Figure 4 were evolved with tuneable parameters *λ*_1_ = 2.5, *λ*_2_ = 1. The area overlap measure of mass B was greater than 0.95; however, the percentage differences in boundary moments were above 18%. For masses with distinct or well-defined margins, similar segmentation results are expected and this is confirmed with a mean area overlap measure of 0.96 ± 0.03 as shown in Table 6. For this category of masses, the mean value of %Δ*F*_1_ was 8.9% (range 0.3–25.0%); of %Δ*F*_2_, 8.6% (range 2.1–33%); and of %Δ*F*_3_, 14.1% (range 0.9–53.0%). The mean Euclidean distance between the Fourier descriptors of the segmented areas was 0.05 ± 0.02 and the mean values of percentage changes of shape convexity and rectangularity were 4.5% (range 0.07–17.2%) and 5.7% (range 0.04−14.9%), respectively. The values for the mean percentage differences in shape convexity and rectangularity were almost 50% less than those from boundary moments. This group presented a small percentage change in shape convexity and shape rectangularity and also a small mean Euclidean distance of the Fourier descriptors as compared to the previous group due to segmentation results having relatively similar shapes. For these groups of masses, shape-based descriptors derived from final contours of tuneable parameters *λ*_1_ = 1, *λ*_2_ = 1 were less sensitive to changes in the location of the initial level set contours. Table 6 shows that the mean percentage differences of the shape convexity and shape rectangularity are less than the values for the boundary moments. Table 7 illustrates similar trends with the selective local or global segmentation model; however, the Jaccard similarity indices of the Chan-Vese segmentation model for this group of masses were greater than values obtained by using the selective local or global segmentation model.

The evaluation metrics of shape-based descriptors of both groups of masses were combined and assessed with Bland-Altman plots to investigate the intermethod agreement between placements of the initial level set contours. Each Bland-Altman plot was evaluated within a 95% confidence interval as the limits of agreement.

**Figure 5:**
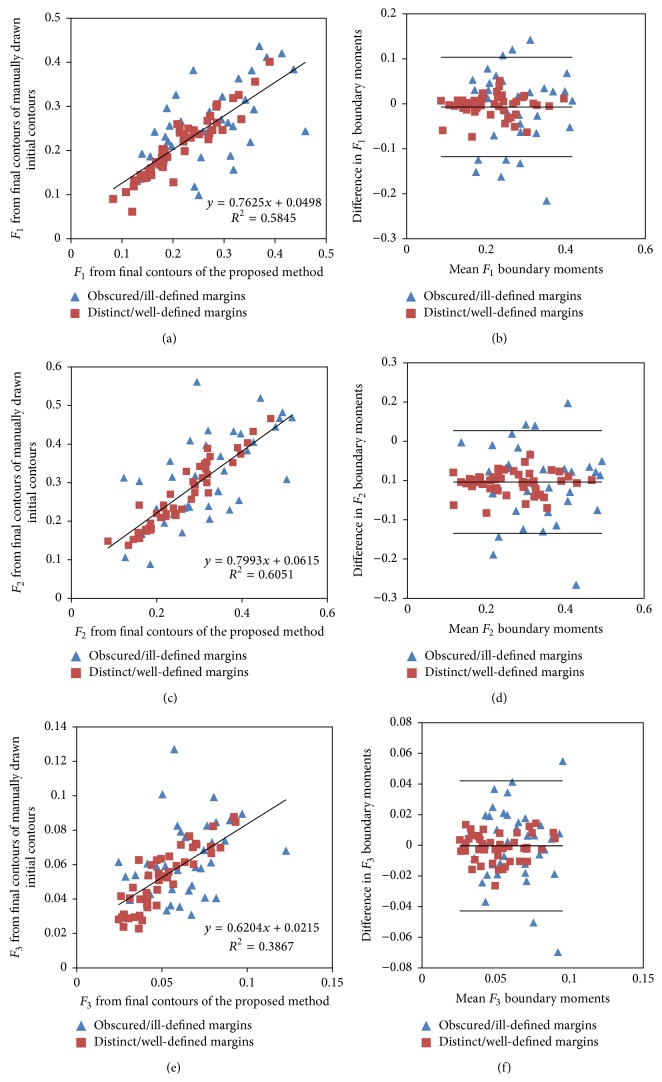
Linear regression plots ((a), (c), and (e)), along with Bland-Altman plots ((b), (d), and (f)), of boundary moments *F*_1_, *F*_2_, and *F*_3_, respectively, for tuneable parameters *λ*_1_ = 2.5, *λ*_2_ = 1.

**Figure 6:**
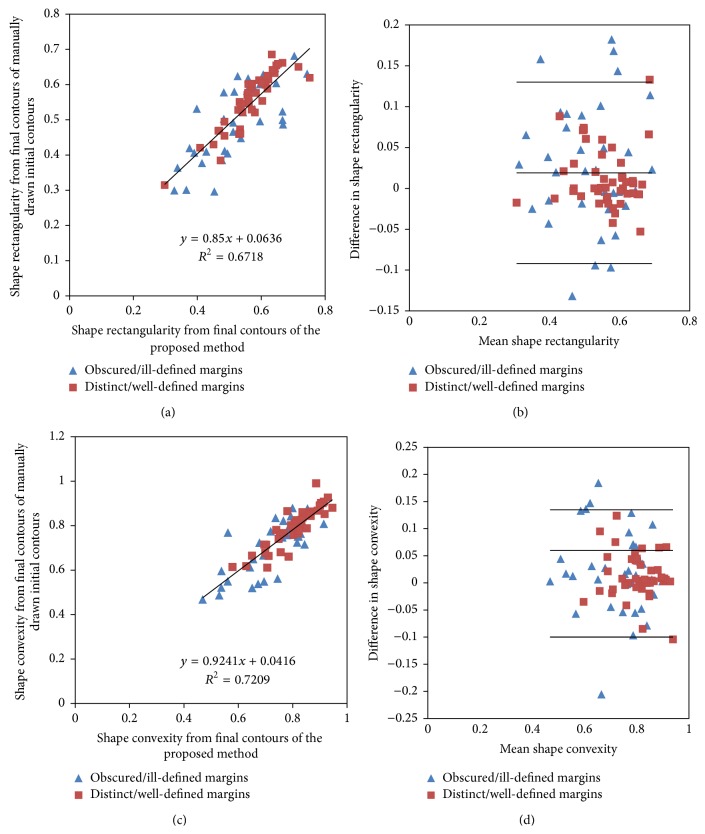
Linear regression plots ((a) and (c)) along with associated Bland-Altman plots ((b) and (d)) of shape rectangularity (SR) and shape convexity (SC), respectively, for tuneable parameters *λ*_1_ = 2.5, *λ*_2_ = 1.

Figures 5 and 6 illustrate the linear regression plots of boundary moments, shape rectangularity, and shape convexity with their associated Bland-Altman plots with the Chan-Vese segmentation method. The Pearson correlation analysis indicated good correlations between the shape-based descriptors: shape rectangularity (*r* = 0.82) and shape convexity (*r* = 0.82) resulting from the final contours of the proposed and manual methods as compared to boundary moments *F*_1_  (*r* = 0.76), *F*_2_  (*r* = 0.77), and *F*_3_  (*r* = 0.68). The selective local or global segmentation method gave higher correlation coefficients for these shape descriptors.  Table 8 shows the summary results of the linear regression analysis of shape-based descriptors for these masses and their variation with tuneable parameters. *p* values indicated that the correlations of shape-based descriptors derived from these methods were statistically significant (*p* < 0.0001). The strength of the linear relationship (*r*) between the descriptors derived from these methods depends on the values of tuneable parameters, *λ*_1_ and *λ*_2_, for the Chan-Vese model. For this database of masses, the correlation coefficients of descriptors obtained with tuneable parameters *λ*_1_ = 1 and *λ*_2_ = 1 were higher than those with parameters *λ*_1_ = 2.5 and *λ*_2_ = 1; however, this does not imply that tuneable parameters *λ*_1_ = 1 and *λ*_2_ = 1 will provide higher values of similarity measures when segmentation results are compared with segmentation outcomes of expert radiologists. Overall, the performance of the selective local or global segmentation model was similar to the performance of the Chan-Vese segmentation model for this database of direct digital mammographic masses.

The difference plots in Figures 5 and 6 show that differences in shape-based features for masses with distinct or well-defined margins are scattered very close to the central bias line as compared to masses with obscured, or ill-defined, margins, thus indicating that the magnitude of differences in shape-based descriptors due to changes in the placement of the initial level set contours depends on the mass margin characteristics. Other researches have reported the variation of segmentation accuracy with the characteristic of the mass margins for a given segmentation algorithm [41]. The correlations (*r*s < 0.06, *p* > 0.05) between differences in shape-based descriptors due to changes in the placement of the initial level set contours and the average magnitude of descriptors from both algorithms were very poor and they were not significantly different from zero.

In general, the mean area overlap measure of the combined categories was 0.89 ± 0.02, the mean Euclidean distance between the Fourier descriptors was 0.07 ± 0.05, and moreover, in the Bland-Altman plots, the differences in shape-based descriptors of 90% of these masses are within the limits of agreement; therefore the interplacement agreement of the initial level set contours based on these descriptors is acceptable. However, both segmentation methods illustrated large variation in boundary moments as compared to shape-based descriptors such as shape convexity, shape rectangularity, and Euclidean distance of the Fourier descriptors. Hence, boundary moments should be utilized with caution because they exhibit large percentage differences.

Interobserver variability amongst radiologists and intermethod variability in delineating masses in mammography translate to differences in shape-based feature vectors. The magnitude of these differences should however not be so large as to compromise the interclass separability measures and hence the classification accuracies of shape-based binary classifiers. This can be achieved if these feature vectors show a certain degree of robustness to interobserver and intermethod variability in segmented masses.

## 5. Conclusion

We have investigated and quantified the variations in shape-based features in segmentation outcomes due to differences in the location of the initial level set contour for mass lesion segmentation in direct digital mammography. The Chan-Vese segmentation method and the active contours with selective local or global segmentation model presented similar results. The results show that the magnitude of these variations expressed as area overlap measures and percentage differences in shape-based features depend on the characteristics of the mass margins and the choice of tuneable parameters. For masses with distinct or well-defined margins, percentage differences are reduced as compared to those with ill-defined or obscured margins for both segmentation algorithms. The mean percentage differences in boundary moments and their ranges were large as compared to those of shape convexity and shape rectangularity, even though the area overlaps measures were within acceptable values. The influences of these variations on the classification accuracy of shape-based binary classifiers will depend on the magnitude of the interclass separability measures; however, large fluctuations in these values for the same mass are undesirable. Finally, we concluded that boundary moments are sensitive to the placement of initial level set contours while Fourier descriptors, shape convexity, and shape rectangularity exhibit a certain degree of robustness to changes in the location of the initial level set contours.

## Figures and Tables

**Table 1:** Comparison of mean area overlap measures of masses with characterized margins due to changes in the location of the initial level set contour with cited interobserver variability amongst radiologists and with mean area overlap measures between radiologists and other segmentation methods in boundary delineation.

	Characteristics of mass lesion margins	Mean area overlap measures due to interobserver variability amongst radiologists	Mean area overlap measures between radiologists and other segmentation methods	Mean area overlap measures due to the placement of the initial level set contours in this study
Sahiner et al. [17]	—	0.76 ± 0.13	0.74 ± 0.13	

Tao et al. [18]	Ill-defined and spiculated		0.69 ± 0.16	

Xu et al. [19]	—		0.72 ± 0.13	

Rahmati et al. [20]	—		0.87 ± 0.05	

Pereira et al. [21]	—		0.79 ± 0.08	

This study (*λ*_1_ = 2.5, *λ*_2_ = 1)	Obscured/ill-defined with low signal areas within			0.81 ± 0.01

This study (*λ*_1_ = 2.5, *λ*_2_ = 1)	Distinct/well-defined			0.96 ± 0.03

This study (*λ*_1_ = 1, *λ*_2_ = 1)	Obscured/ill-defined with low signal areas within			0.87 ± 0.13

This study (*λ*_1_ = 1, *λ*_2_ = 1)	Distinct/well-defined			0.95 ± 0.06

This study (*α* = 5)	Obscured/ill-defined with low signal areas within			0.86 ± 0.09

This study (*α* = 5)	Distinct/well-defined			0.91 ± 0.04

**Table 2:** Evaluation metrics for differences in segmented areas (JSC) and boundary moments (%Δ*F*_1_, %Δ*F*_2_, and %Δ*F*_3_), due to changes in the location of the initial level set contours evolved with tuneable parameters λ_1_ = 2.5, λ_2_ = 1 for masses in Figure 3.

Masses	JSC	*F*_1_	*F*_2_	*F*_3_	Method	%Δ*F*_1_	%Δ*F*_2_	%Δ*F*_3_
A	0.83	0.3186	0.3715	0.0530	Manual	68.4	67.1	59.8
		0.1563	0.1848	0.0286	Proposed			

B	0.77	0.3417	0.4264	0.0846	Manual	8.1	7.2	3.5
		0.3152	0.3969	0.0817	Proposed			

C	0.78	0.2691	0.3269	0.0578	Manual	9.7	3.1	23.0
		0.2442	0.3170	0.0728	Proposed			

D	0.84	0.2505	0.3120	0.0615	Manual	87.0	86.8	86.0
		0.0986	0.1231	0.0245	Proposed			

E	0.71	0.2715	0.3300	0.0585	Manual	6.0	8.1	17.6
		0.2882	0.3580	0.0698	Proposed			

F	0.89	0.2969	0.3826	0.0857	Manual	8.2	7.4	4.8
		0.3224	0.4122	0.0899	Proposed			

G	0.87	0.1835	0.2168	0.0333	Manual	1.7	9.9	45.3
		0.1866	0.2394	0.0528	Proposed			

**Table 3:** Variation in Euclidean distances between Fourier descriptors (DF), percentage differences in shape convexity (%ΔSC), and shape rectangularity (%ΔSR) due to changes in the location of the initial level set contours evolved with tuneable parameters *λ*_1_ = 2.5, *λ*_2_ = 1 for masses in Figure 3.

Masses	DF	SC	SR	Method	%ΔSC	%ΔSR
A	0.16	0.7152	0.4861	Manual	16.5	31.5
		0.8439	0.6681	Proposed		

B	0.10	0.5373	0.3762	Manual	22.4	9.6
		0.6731	0.4143	Proposed		

C	0.08	0.5610	0.3845	Manual	28.2	21.6
		0.7450	0.4774	Proposed		

D	0.13	0.8071	0.6301	Manual	12.5	16.6
		0.9143	0.7440	Proposed		

E	0.06	0.7737	0.5017	Manual	16.7	3.8
		0.9143	0.4830	Proposed		

F	0.05	0.5955	0.4188	Manual	10.1	10.9
		0.5383	0.3755	Proposed		

G	0.03	0.7899	0.5297	Manual	1.7	8.8
		0.8036	0.5786	Proposed		

**Table 4:** Evaluation metrics for differences in segmented areas (JSC) and boundary moments (%Δ*F*_1_, %Δ*F*_2_, and %Δ*F*_3_), due to changes in the location of the initial level set contours evolved with tuneable parameters *λ*_1_ = 2.5, *λ*_2_ = 1 for masses in Figure 4.

Masses	JSC	*F*_1_	*F*_2_	*F*_3_	Method	%Δ*F*_1_	%Δ*F*_2_	%Δ*F*_3_
A	0.98	0.0827	0.1051	0.0224	Manual	29.0	31.2	39.4
		0.1107	0.1440	0.0334	Proposed			

B	0.97	0.2084	0.2783	0.0699	Manual	21.9	21.1	18.7
		0.2597	0.3440	0.0843	Proposed			

C	0.99	0.1489	0.1785	0.0295	Manual	6.1	2.6	13.6
		0.1401	0.1739	0.0338	Proposed			

D	0.98	0.2130	0.2568	0.0437	Manual	12.0	9.0	6.6
		0.2402	0.2811	0.0409	Proposed			

E	0.98	0.1080	0.1376	0.0296	Manual	2.2	2.9	5.9
		0.1057	0.1336	0.0279	Proposed			

F	0.94	0.1888	0.2192	0.0304	Manual	2.0	1.3	19.3
		0.1851	0.2220	0.0369	Proposed			

G	0.94	0.2971	0.3676	0.0705	Manual	18.7	12.4	10.9
		0.2463	0.3248	0.0786	Proposed			

**Table 5:** Variation in Euclidean distances between the Fourier descriptors (DF), percentage differences in shape convexity (%ΔSC), and shape rectangularity (%ΔSR) due to changes in the location of the initial level set contours evolved with tuneable parameters *λ*_1_ = 2.5, *λ*_2_ = 1 for masses in Figure 4.

Masses	DF	SC	SR	Method	%ΔSC	%ΔSR
A	0.0368	0.6612	0.4619	Manual	17.1	14.5
		0.7852	0.5339	Proposed		

B	0.0624	0.6637	0.4596	Manual	7.0	14.4
		0.7116	0.5307	Proposed		

C	0.0377	0.8894	0.6148	Manual	1.1	1.2
		0.8995	0.6224	Proposed		

D	0.0762	0.8508	0.6093	Manual	0.0	0.0
		0.8508	0.6093	Proposed		

E	0.0216	0.9267	0.6544	Manual	0.3	1.1
		0.9295	0.6472	Proposed		

F	0.0298	0.8606	0.6024	Manual	2.9	5.2
		0.8360	0.5716	Proposed		

G	0.0717	0.6612	0.4619	Manual	17.1	14.5
		0.7852	0.5339	Proposed		

**Table 6:** Mean values for the Jaccard similarity coefficient (JSC) and the Euclidean distances of the masses. The mean values and ranges of percentage differences in boundary moments (%Δ*F*_1_, %Δ*F*_2_, and %Δ*F*_3_), percentage differences in shape convexity (%ΔSC), and percentage differences in shape rectangularity (%ΔSC) for the masses, labelled as groups with predefined margin characteristics and also a group with arbitrary margin characteristics, due to changes in the location of the initial level set contours evolved with tuneable parameters λ_1_ = 2.5, λ_2_ = 1 and λ_1_ = 1, λ_2_ = 1.

Margin characteristics	Obscured/ill-defined margins		Distinct/well-defined margins		Unlabelled margins	
Tuneable parameters	*λ*_1_ = 2.5, *λ*_2_ = 1	*λ*_1_ = 1, *λ*_2_ = 1	*λ*_1_ = 2.5, *λ*_2_ = 1	*λ*_1_ = 1, *λ*_2_ = 1	*λ*_1_ = 2.5, *λ*_2_ = 1	*λ*_1_ = 1, *λ*_2_ = 1
Average JSC	0.81 ± 0.01	0.87 ± 0.13	0.96 ± 0.03	0.95 ± 0.06	0.89 ± 0.02	0.92 ± 0.09
Average DF	0.09 ± 0.05	0.05 ± 0.04	0.05 ± 0.02	0.04 ± 0.02	0.07 ± 0.05	0.05 ± 0.03
Mean of %Δ*F*_1_	23.9%	17.5%	8.9%	11.4%	16.4%	14.5%
Range of %Δ*F*_1_	1.0–87.0%	0–62.6%	0.3–25.0%	0–24.4%	1.0–87.0%	0–62.6%
Mean of %Δ*F*_2_	24.5%	17.2%	8.6%	9.6%	16.6%	13.4%
Range of %Δ*F*_2_	1.7–86.8%	0–59.1%	2.1–33%	0–46.0%	1.7–86.8%	0–59.1%
Mean of %Δ*F*_3_	32%	20.1%	14.1%	13.4%	23.1%	16.8%
Range of %Δ*F*_3_	1.4–86.0%	0–80.9%	0.9–53.0%	0–54.0%	0–86.0%	0–80.9%
Mean of %ΔSC	8.3%	2.4%	4.5%	2.9%	6.4%	2.7%
Range of %ΔSC	0.0–28.1%	0–21.0%	0.07–17.2%	0.2–13.9%	0.3–28.1%	0–21.0%
Mean of %ΔSR	11.7%	7.6%	5.7%	4.3%	8.7%	5.9%
Range of %ΔSR	0.1–42.0%	0–38.9%	0.04–14.9%	0–21.9%	0.1–42.0%	0–38.9%

**Table 7:** Mean values for the Jaccard similarity coefficient (JSC) and the Euclidean distances of the masses. The mean values and ranges of percentage differences in boundary moments (%Δ*F*_1_, %Δ*F*_2_, and %Δ*F*_3_), percentage differences in shape convexity (%ΔSC), and percentage differences in shape rectangularity (%ΔSC) for the masses, labelled as groups with predefined margin characteristics and also a group with arbitrary margin characteristics, due to changes in the location of the initial level set contours evolved with the selective local or global segmentation model with tuneable parameter *α* = 5.

Margin characteristics	Obscured/ill-defined margins	Distinct/well-defined margins	Unlabelled margins
Average JSC	0.86 ± 0.09	0.91 ± 0.04	0.89 ± 0.07
Average DF	0.05 ± 0.04	0.04 ± 0.03	0.05 ± 0.04
Mean of %Δ*F*_1_	15.1%	11.1%	13.1%
Range of %Δ*F*_1_	0–74%	0–38%	0–74%
Mean of %Δ*F*_2_	15.4%	13.4%	14.4%
Range of %Δ*F*_2_	0–67.5%	0–44.2%	0–67.5%
Mean of %Δ*F*_3_	23.5%	15.1%	19.3%
Range of %Δ*F*_3_	0–52%	0–41.1%	0–52%
Mean of %ΔSC	10.2%	8.7%	9.5%
Range of %ΔSC	0–30%	0–25.1%	0–30%
Mean of %ΔSR	11.7%	9.2%	10.5%
Range of %ΔSR	0–30%	0–28%	0–30%

**Table 8:** Summary results of linear regression analysis for tuneable parameters *λ*_1_ = 2.5, *λ*_2_ = 1 and *λ*_1_ = 1, *λ*_2_ = 1 and the selective local or global segmentation method.

	Tuneable parameters						Selective local or global segmentation method		
	*λ*_1_ = 2.5, *λ*_2_ = 1			*λ*_1_ = 1, *λ*_2_ = 1			*α* = 5		
	Slope	*r*	*p* value	Slope	*r*	*p* value	Slope	*r*	*p* value
*F*_1_	0.76	0.76	<0.0001	0.83	0.81	<0.0001	0.69	0.81	<0.0001
*F*_2_	0.79	0.77	<0.0001	0.72	0.80	<0.0001	0.75	0.86	<0.0001
*F*_3_	0.62	0.68	<0.0001	0.75	0.74	<0.0001	0.7	0.74	<0.0001
SC	0.85	0.82	<0.0001	0.93	0.88	<0.0001	0.88	0.83	<0.0001
SR	0.92	0.82	<0.0001	0.82	0.88	<0.0001	0.85	0.94	<0.0001
